# Microbial signatures and enterotype clusters in fattening pigs: implications for nitrogen utilization efficiency

**DOI:** 10.3389/fmicb.2024.1354537

**Published:** 2024-04-10

**Authors:** Naomi Sarpong, Jana Seifert, Jörn Bennewitz, Markus Rodehutscord, Amélia Camarinha-Silva

**Affiliations:** ^1^Institute of Animal Science, University of Hohenheim, Stuttgart, Germany; ^2^HoLMiR - Hohenheim Center for Livestock Microbiome Research, University of Hohenheim, Stuttgart, Germany

**Keywords:** microbiota, nitrogen utilization efficiency, sustainability, health, pig, biomarker, phase feeding, fattening

## Abstract

As global demand for pork continues to rise, strategies to enhance nitrogen utilization efficiency (NUE) in pig farming have become vital for environmental sustainability. This study explored the relationship between the fecal microbiota, their metabolites, and NUE in crossbreed fattening pigs with a defined family structure. Pigs were kept under standardized conditions and fed in a two-phase feeding regime. In each phase, one fecal sample was collected from each pig. DNA was extracted from a total of 892 fecal samples and subjected to target amplicon sequencing. The results indicated an influence of sire, sampling period (SP), and sex on the fecal microbiota. *Streptococcus* emerged as a potential biomarker in comparing high and low NUE pigs in SP 1, suggesting a genetic predisposition to NUE regarding the fecal microbiota. All fecal samples were grouped into two enterotype-like clusters named cluster LACTO and cluster CSST. Pigs’ affiliation with enterotype-like clusters altered over time and might be sex-dependent. The stable cluster CSST demonstrated the highest NUE despite containing pigs with lower performance characteristics such as average daily gain, dry matter intake, and daily nitrogen retention. This research contributes with valuable insights into the microbiome’s role in NUE, paving the way for future strategies to enhance sustainable pig production.

## 1 Introduction

The most recent projections from OECD/FAO anticipate that global pork consumption will ascend to over 129.3 million tons by 2032 due to increasing population and rising incomes (OECD-FAO, 2023). Pork is an excellent protein source and contains minerals and vitamins (e.g., iron and thiamine) that can benefit human nutrition (Cobos and Díaz, 2015). Nevertheless, intensive pig production is frequently linked with environmental pollution, wherein nitrogen (N) is a significant contributor (Aarnink and Verstegen, 2007). A growing pig retains only around 30% of the ingested N (Shirali et al., 2012), while the remainder is excreted via urine and feces (Flachowsky and Kamphues, 2012). Regardless of the production stage, the average proportion of excretion via urine is usually markedly higher (45–50%) than via feces (17–19%; Dourmad et al., 1999). The excreted N after microbial transformations can pollute the atmosphere in the form of ammonia (NH_3_), nitrous oxide (N_2_O), and N oxide (NO_x_) emissions, and the ground and surface waters through leaching and runoff of N compounds such as nitrate (${\text{NO}}_{3}^{-}$; Leip et al., 2014).

Nitrogen utilization efficiency (NUE) indicates the percentage of ingested N retained in the body. In sustainable pig production, maximization of NUE is mandatory (Millet et al., 2018). Precision feeding with diets based on the concept of ideal protein (van Milgen and Dourmad, 2015) leads to an increased NUE compared to conventional phase-feeding (Andretta et al., 2016). However, this is challenging to implement under practical husbandry conditions. Consequently, opportunities beyond feeding strategies are needed. A long-term approach could be to increase the NUE at the animal level through genetic improvement of the population (Oenema and Tamminga, 2005). A valuable tool for identifying pigs with an increased NUE is the implementation of diets with restriction of growth-determining amino acid (AA) concentration, such as lysine (GfE, 2008). Blood urea nitrogen (BUN) can be used to predict urinary N excretion in pigs of comparable production stages within a study (Kohn et al., 2005), and a negative correlation between BUN and NUE in growing pigs is described (Tran-Thu, 1975; Berschauer, 1977; Whang et al., 2003; Schmid et al., 2024). BUN concentration appears to be a useful auxiliary feature for determining the NUE, in groups with large numbers of animals that have a limited lysine supply (Berghaus et al., 2023).

The absorption and utilization of proteins in pigs is influenced by the AA availability in the diet (Mi et al., 2023). Different protein sources exhibit various patterns of AAs release, that are influenced by the solubility and digestibility of the protein (Wang et al., 2021). A faster and more synchronized release of AAs leads to reduced N losses and improved NUE (Wang et al., 2021). Dietary and endogenous N sources that escape endogenous enzymatic digestion can be fermented by the gut microbiota, mainly in the large intestine, due to its larger cell population and slower passage rate of digesta than in the small intestine (Williams et al., 2001; Zhang et al., 2020). Volatile fatty acids (VFAs) and NH_3_ are the main end-products (Zhang et al., 2020). In particular, branched-chain fatty acids, e.g., iso-butyrate and iso-valerate, serve as markers since they solely occur in proteolytic fermentation (Mortensen and Clausen, 1996). Acetate, propionate, and butyrate are generated from AA, but in much larger quantities from fermentable carbohydrates (Cummings et al., 1987; Smith and Macfarlane, 1997). A few studies showed the influence of dietary protein content on the microbiota composition (Fan et al., 2017; Spring et al., 2020; Tao et al., 2021). A study with diets varying in protein levels (standardized ileal digestibility of 17.5% vs. 14.9%) and protein sources (casein, corn gluten meal or mix diet) revealed changes in the microbiota community. The diets with lower standardized ileal digestibility resulted in greater microbial diversity and an increased abundance of *Lactobacillus* in the jejunum. Casein or mix diet as protein source led to a higher microbial diversity in the jejunum than the diet with corn gluten meal through the equalized release of AAs (Mi et al., 2023). A dietary crude protein (CP) content of 12% led to lower growth performance in pigs combined with lower abundance of *Prevotella*, while *Christensenellaceae*, *Aligiphilus*, and *Algoriphagus* were more abundant in the pig feces (Spring et al., 2020). Pigs with low body weight (BW) were associated with lower fecal microbial diversity and higher levels of opportunistic pathogenic bacteria such as *Anaerotruncus* and *Bacteroides* (Han et al., 2017).

Enterotyping, firstly described in microbiome research with humans (Arumugam et al., 2011) and later applied to pig studies (Mach et al., 2015; Ramayo-Caldas et al., 2016; Lu et al., 2018; Yang et al., 2018; Ke et al., 2019; Le Sciellour et al., 2019; Ma et al., 2022), involves clustering to decrease the complexity of the fecal microbiota (Costea et al., 2018). Enterotype-like clusters are defined as groups of samples sharing similar bacterial composition (Arumugam et al., 2011). In pigs, enterotypes have been associated with a variety of factors, including performance (Mach et al., 2015; Lu et al., 2018; Ke et al., 2019; Le Sciellour et al., 2019; Ma et al., 2022). Previous studies showed that the affiliation of a pig to an enterotype can change over time (Mach et al., 2015; Lu et al., 2018; Le Sciellour et al., 2019). However, the reasons behind these shifts remain largely unexplored but are of significant interest. In addition, the relationship between the pigs’ enterotype affiliation and NUE has not yet been explored.

Although optimization of animal husbandry, genetics, and feeding strategies have significantly improved feeding efficiency in recent decades (Reyer et al., 2020), there is still a lack of studies addressing the microbial communities in pigs with varying predispositions to NUE. The study aimed to reveal the interrelationship between the fecal microbiota of fattening pigs with a defined family structure, their produced metabolites, and NUE.

## 2 Materials and methods

### 2.1 Description of the animal experiment, diet and sampling

The experiment was carried out for 2.5 years (2018–2021) at the Agriculture Experimental Station of the University of Hohenheim following German Animal Welfare Legislation after approval of the Regierungspräsidium Tübingen, Germany (Project no. HOH52/18 TE). Supplementary Figure S1 gives an overview of the experimental design. In total 508 crossbreed pigs (German Landrace×Piétrain) with defined family structures were kept under standardized conditions and investigated into 21 cohorts. A detailed description of the experimental design and procedures is given in Berghaus et al. (2023). Briefly, at the 11th week of life, cohorts were moved into the experimental barn for individual housing. Depending on the cohort size, for both sexes the heaviest animals and those closest to the average litter weight were selected. A two-phase feeding regime was implemented over the experimental period. The starter phase was administered from the 11th week of life when the pigs were kept separately. The feed transition to the grower phase occurred in the 14th week of life. In both phases, the diet was based on barley, wheat, and soybean meal (Supplementary Table S1). The proportions of the ingredients were adjusted to meet 90% of the demand for prececal digestible lysine in the prevailing phase in accordance with the Gesellschaft für Ernährungsphysiologie recommendations (GfE, 2008). This restriction allowed the pigs to show their full genetic potential for NUE. The following data were recorded to calculate the respective performance characteristics: BW to calculate average daily gain (ADG); the amount of feed administered and feed refused to estimate dry matter intake (DMI); gain to feed ratio (G:F) and average daily nitrogen intake (DNI). Two sampling periods (SPs) were executed for each cohort. In the 13th week of life, the first sampling period (SP 1) was conducted, followed by a second sampling period (SP 2) in the 16th week of life. The BW was 40.5 ± 4.7 kg in SP 1 and 60.3 ± 7.0 kg in SP 2 (mean ± standard deviation (SD)). At the end of the experiment, the BW was 96.0 ± 9.5 kg. A subgroup of pigs (*n* = 48) were randomly selected throughout the experimental period for N balance measurement in combination with stable isotope tracer technique as described in detail in Berghaus et al. (2023).

Each SP was conducted during five consecutive days following the same procedure: during the time range from the second to the fourth day (14,30–16,00 daily), a single fecal sample was collected once from each pig immediately after defecation. Samples were kept on ice and stored at −80°C until DNA and VFAs were extracted. Fifty animals were excluded from the analysis due to an experimental break because of the pandemic. Five further animals received antibiotic treatment and were excluded from the study. The fecal samples of in total 453 pigs were in both SPs available and were used for the purpose of this study.

On all days of the respective SP, fecal samples were collected from each pig to measure total N and dry matter (DM; Berghaus et al., 2023). Blood samples were collected to determine the BUN level, serum cortisol (Cor), and serum insulin-like growth factor 1 (IGF-1). For the pigs subjected to balance measurement, daily urinary N excretion and daily fecal N excretion were determined in addition to DNI. These values and blood metabolite concentrations were used to estimate daily nitrogen retention (DNR) for all pigs by multiple regressions and further calculate NUE (Berghaus et al., 2023). Those pigs (*n* = 446) whose fecal samples in both SPs passed the bioinformatic quality control described below, were divided into two groups (low and high) within each SP based on their NUE. The group NUE_Low_ contained pigs with values belonging to the 25% quantile. Pigs belonging to the 75% quantile were considered in the group NUE_High_.

### 2.2 VFA determination

Each fecal sample of 23.8 ± 8.74 g (mean ± SD) was diluted with 17.74 ± 7.01 g of ultrapure water to achieve homogeneity. Two aliquots with a weight of 4 g were taken from each sample and acidified with 2.5 mL sulfuric acid (5 N H_2_SO_4_). The 80 mM 2-methylvaleric acid in 50% formic acid was used as an internal standard. After a second homogenization step, the samples were frozen in Erlenmeyer flasks under continuous movement using a − 30°C ethanol bath. The distillation of the non-dissociated fatty acids was carried out under vacuum. To measure the quantity of acetate, propionate, iso-butyrate, butyrate, iso-valerate, and valerate in mmol/kg fresh matter, a gas chromatograph (Hewlett-Packard 6,890; Agilent) provided with a flame ionization detector and a fused silica capillary column (HP-FFAP; 25 m by 0.32 mm with a film thickness of 0.5 μm; HP 7683; Agilent) was used, as reported by Wischer et al. (2013).

### 2.3 DNA extraction and sample preparation for target sequencing

Microbial DNA was extracted from 250 mg of each fecal sample (*n* = 906) using the commercial FastDNA^™^ SPIN Kit for Soil (MP Biomedical, Solon, OH, United States). DNA was quantified with a NanoDrop 2000 Spectrophotometer (Thermo Fisher Scientific, Waltham, MA, United States) and stored at −20°C. Amplicon sequencing libraries preparation of the V1-V2 region of the 16S rRNA gene was carried out with PrimeSTAR^®^ HS DNA Polymerase kit (TaKaRa, Beijing, China) following the method described in Kaewtapee et al. (2017). Briefly, 1 μL of DNA was added for the first PCR, in a 20-μl reaction with 0.2 μL of PrimeSTAR HS DNA polymerase and 0.5 μL of each primer. The second PCR, which used 1 μL of the first PCR as a DNA template, ran in a total volume of 50 μL. An initial denaturation at 95°C for 3 min was followed by 15 cycles (first PCR) or 20 cycles (second PCR) of denaturation at 98°C for 10 s, subsequent annealing at 55°C for 10 s, extension step at 72°C for 45 s and a final extension for 2 min at 72°C. Derived amplicons were inspected by agarose gel electrophoresis, purified, and normalized using SequalPrep Normalization Kit (Invitrogen Inc., Carlsbad, CA, United States). Samples were sequenced using 250 base pairs (bp) paired-end sequencing chemistry on an Illumina Novaseq 6,000 platform.

### 2.4 Bioinformatical analysis

After demultiplexing Fastq files with Sabre, they were further processed with Qiime2 (v.2021.4; Bolyen et al., 2019). Reads were trimmed using the q2-cutadapt plugin (Martin, 2011). The q2-dada2 plugin was used to identify amplicon sequence variants (ASVs) through quality filtering, error correction, dereplication, and merging of reads (Callahan et al., 2016). ASVs with a sequence length of less than 100 bp and/or that occurred in fewer than ten samples were removed. Sklearn-based classifiers (Pedregosa et al., 2011) were generated with RESCRIPt (Robeson et al., 2021) using the Silva SSU-rRNA database (v.138.1, 16S 99%; Quast et al., 2013). These were used for the taxonomic assignment of ASVs using VSEARCH (Rognes et al., 2016). Samples containing less than 50 ASVs and/or having ASVs with a sequence length of less than 200 bp were removed. Reads from organelles and unassigned sequences were deleted from the analysis. Twelve samples were excluded from further analysis because of the fewer reads. Two further samples were excluded because only one SP has enough reads after quality filtering.

Illumina amplicon data were analyzed using R version 4.3.1 (2023-06-16; Ihaka and Gentleman, 1996). The Shannon diversity index was calculated with “vegan” to assess the α-diversity in the samples (Oksanen et al., 2022). The total number of reads was standardized per sample and a sample dissimilarity matrix was created using the Bray–Curtis dissimilarity coefficient. Permutation analysis of variance (PERMANOVA) using the “adonis” function of the package “vegan” was used to compare microbial communities associated with SPs, sex and different sires (Oksanen et al., 2022). Further PERMANOVAs were calculated within each SP to examine whether the microbial communities differed between the NUE_High_ and NUE_Low_ groups. For visualization of ordination and clustering of samples, non-metric multidimensional scaling (NMDS) was plotted using “ggplot2” (Wickham, 2016). The barplot for the relative abundance of the microbial composition at the genus level was generated with “ggplot2.” If the PERMANOVA revealed a significant difference between the NUE_High_ and NUE_Low_ groups, a linear differential abundance analysis (LinDA) model was applied to identify the genera that were associated the differences in the respective SP (Zhou et al., 2022). Therefore, the relative abundance data at the genus level was run with the function “linda” of the package “MicrobiomeStat.” The default settings were kept. LinDA was visualized as a volcano plot with the “linda.plot” function.

### 2.5 Cluster analysis of the fecal microbiota

Enterotypes were assigned following the methodology from Arumugam et al. (2011) to identify groups of samples with similar bacterial composition. The Jensen–Shannon divergence matrix was calculated from the relative genus abundance data (unclassified genera were excluded) and partitioning around medoids clustering algorithm was applied using “cluster” (Maechler et al., 2022). To assess the optimal number of clusters (corresponding to enterotypes), the Calinski-Harabasz Index was calculated with “clusterSim” (Walesiak and Dudek, 2020) and the average silhouette width was determined with “factoextra” (Alboukadel and Mundt, 2020). Principal coordinates analysis (PCoA) was carried out and visualized with the function “dudi.pco” of the package “ade4” (Dray and Dufour, 2007). Using between-class analysis, the enterotype-like clusters were named according to the genus with the respective highest taxon weight. To investigate the relationship between these genera and the other representatives within each enterotype-like cluster, a co-occurrence network analysis with “ggraph” was performed (Pedersen, 2021). The nodes represented different genera, and the edges marked positive and negative relations. Genera with a relative abundance >1% were standardized by calculating the Z-scores. This was used as an input for calculating the Spearman correlation coefficients using the “cor” function from the “Stats” package (R Core Team, 2020). Only significant correlations (*p* < 0.0001) with an r > 0.3 or r < −0.3 were considered. Similarity percentage analysis (SIMPER) was run in PRIMER (v.6.1.16, PRIMER-E, Plymouth Marine Laboratory, Plymouth, United Kingdom) at the ASV level to identify the ASVs responsible for the differences between the two enterotype-like clusters (Clarke and Warwick, 2001).

To evaluate the affiliation of pigs to an enterotype-like cluster across both SPs, a manual subgrouping of the samples was performed. Samples from pigs belonging to the same enterotype-like cluster in both SPs were given the index “stable” to the name of the cluster. Those that changed their affiliation to an enterotype-like cluster were named with the cluster of SP 1 and the index “unstable.” For determining the sex distribution, the number of female and male pigs were counted within each subgroup of the enterotype-like clusters.

Beta-diversity analysis was done with all ASVs (unclassified taxa were included) associated with the stable and unstable members of the enterotype-like clusters. PCoA was run with the package “vegan” based on the Bray–Curtis dissimilarity coefficient (Oksanen et al., 2022). The same dissimilarity coefficient was used to calculate PERMANOVA in the way above. Pairwise PERMANOVA of the package “pairwiseAdonis” was used to detect which enterotype-like cluster subgroups differed in microbial composition (Martinez Arbizu, 2020). The relative abundances at the genus level and of the top 15 ASVs determined with SIMPER, were visualized with the package “ggplot2” (Wickham, 2016) to compare the stable and unstable members of the enterotype-like clusters. The representative species for the ASVs were identified using the Basic Local Alignment Search Tool (NCBI Resource Coordinators, 2013). The VFA concentrations, performance characteristics and blood metabolites (all averaged over both SPs) were used to calculate the Z-scores for each stable and unstable enterotype-like cluster and visualized using “ggplot2” (Wickham, 2016).

### 2.6 Functional prediction

The workflow of Tax4Fun2 (v1.1.5) was used to predict the metabolic pathways of the microbial community in each enterotype (Wemheuer et al., 2020). Bacterial genomes were downloaded from the National Center for Biotechnology Information webpage (Sayers et al., 2022) to generate a user-specific reference dataset through 16S rRNA sequence extraction and functional annotation. This reference dataset and those implemented in the package (Ref100 NR) and the ASV table of all samples were used to perform functional prediction. In total 350 Kyoto Encyclopedia of Genes and Genome (KEGG) pathways were identified. PCo plot for the stable and unstable subgroups of the enterotype-like cluster was done based on the Bray–Curtis dissimilarity coefficient with the package “vegan” (Oksanen et al., 2022). PERMANOVA and pairwise PERMANOVA were calculated in the aforementioned way to compare the functional blocks between the subgroups. After filtering for “Amino acid metabolism,” “Carbohydrate metabolism,” “Lipid metabolism,” “Metabolism of other amino acids” and “Energy metabolism” at level 2, 62 KEGG pathways remained. To identify the KEGG pathways that caused the differences between the subgroups of the enterotype-like clusters, the linear discriminant analysis effect size (LEfSe) algorithm approach was applied using the package “microeco” (Liu et al., 2021). The linear discriminant analysis (LDA) score threshold was set to 2. Only the top 30 KEGG pathways were displayed. The corresponding relative abundances of these, prevalent in the subgroups were visualized in a barplot with the package “vegan” (Oksanen et al., 2022).

### 2.7 Statistical analysis

For the comparison of the Shannon diversity index and the relative abundance data at the genus (unclassified genera were included) and ASV level between the stable and unstable members of the enterotype-like clusters, the Kruskal Wallis test with Benjamini-Hochberg (BH) procedure was carried out (Robinson et al., 2022; Wickham et al., 2023). In the case of significance (*p* < 0.05), Wilcoxon test followed by BH procedure was conducted for pairwise comparison (Robinson et al., 2022; Wickham et al., 2023). The same test was used to analyze differences of the Shannon diversity index and the relative abundance data at the genus level (unclassified genera were included) between the enterotype-like clusters, and high and low NUE groups. The Shapiro–Wilk normality test was used to test the normal distribution of VFA concentrations, performance characteristics and blood metabolites (Villasenor Alva and Estrada, 2009). In the case of normal distribution, the homogeneity of variance in the different groups was checked by the function “leveneTest” (Fox and Weisberg, 2018). After running a one-way ANOVA test with the function “aov,” the function “TukeyHSD” was used to make multiple pairwise comparisons between the means of the groups (R Core Team, 2020). To compare the non-normally distributed data, the Kruskal Wallis test and the Wilcoxon test (both with BH procedure) were performed (Robinson et al., 2022; Wickham et al., 2023). Spearman correlation coefficients between genera (average relative abundance higher than 1%, unclassified genera were excluded), VFA concentrations, performance characteristics and blood metabolites (all averaged over both SPs) were calculated within each subgroup of enterotype-like clusters after determining the Z-scores for standardization, as described above. The visualization was done with the R package svglite (Wickham et al., 2023). Only significant correlations (*p* < 0.05) with r > 0.3 or r < −0.3 were included.

## 3 Results

### 3.1 Fecal microbial composition

**Figure 1:**
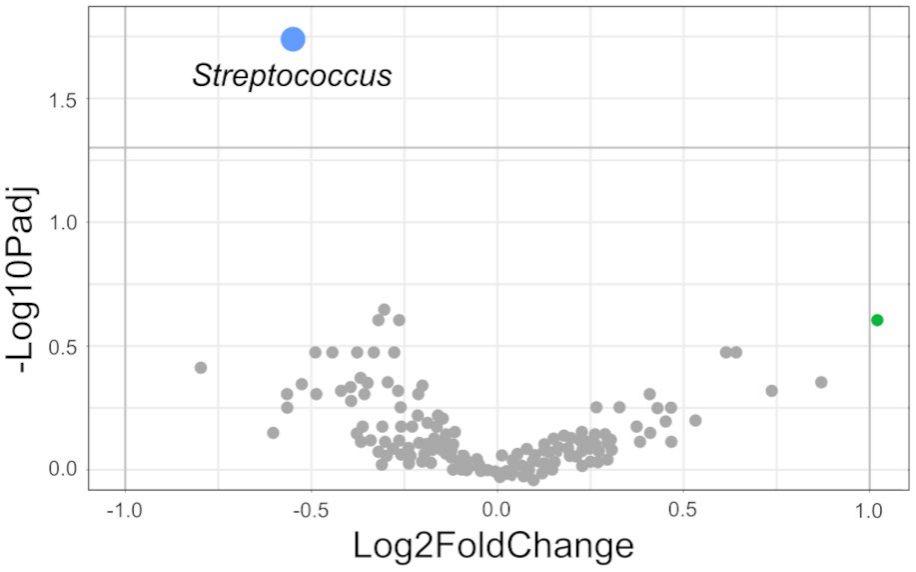
Differential abundant genera (NUE_High_ vs. NUE_Low_ group in SP 1). Genera identified by LinDA and plotted as volcano plot with negative Log10 of Benjamini–Hochberg adjusted *p*-value over Log2FoldChange (LFC). Differential genus with significant differences is marked in blue. Genera without significant differences and LFC ≤ 1 were marked in gray. Differential genus with LFC > 1 and without significant differences was indicated in green (*Libanicoccus*). Negative LFC shows an increased relative abundance in the NUE_High_ group. Positive LFC shows an increased relative abundance in the NUE_Low_ group.

After quality control, sequencing data from 892 fecal samples were used for further analysis. All samples showed an average of 44,204 ± 875 (mean ± standard error (SEM)) reads per sample, and a total of 5,956 ASVs were identified. PERMANOVA revealed that microbial composition in the feces differed between SPs (*p* < 0.001), sex (*p* < 0.001) and sires (*p* < 0.001; Supplementary Table S2). The factor “sire” had the largest impact on the sample grouping (*R^2^* = 0.11), followed by factor “SP” (*R^2^* = 0.03) and “sex” (*R^2^* = 0.009). The groups NUE_High_ and NUE_Low_ included approximately the same number, but different pigs in each SP (SP 1: NUE_High_ = 112 pigs, NUE_Low_ = 111 pigs; SP 2: NUE_High_ = 110 pigs, NUE_Low_ = 109 pigs). Within SP 1, microbial community composition differed between pigs belonging to the NUE_High_ and NUE_Low_ groups (*p* < 0.001; Supplementary Table S3, Figure S2). The sires’ offspring were represented unequally in both groups (Supplementary Table S4). The comparison of the microbial composition between the NUE_High_ and NUE_Low_ groups in SP 2 revealed a trend (*p* = 0.06; Supplementary Table S3). No significant differences were observed between the NUE_High_ and NUE_Low_ groups for the Shannon diversity index (NUE_High_: 4.54; NUE_Low_: 4.58; *p* = 0.58). LinDA revealed *Streptococcus* as a differentially abundant genus for the comparison between NUE_High_ and NUE_Low_ groups (*p* < 0.05; Figure 1). The relative abundance was higher in NUE_High_ (16.4%) than in NUE_Low_ (12.9%) group. All other differentially abundant genera were not significant.

### 3.2 Enterotype-like clusters

**Figure 2:**
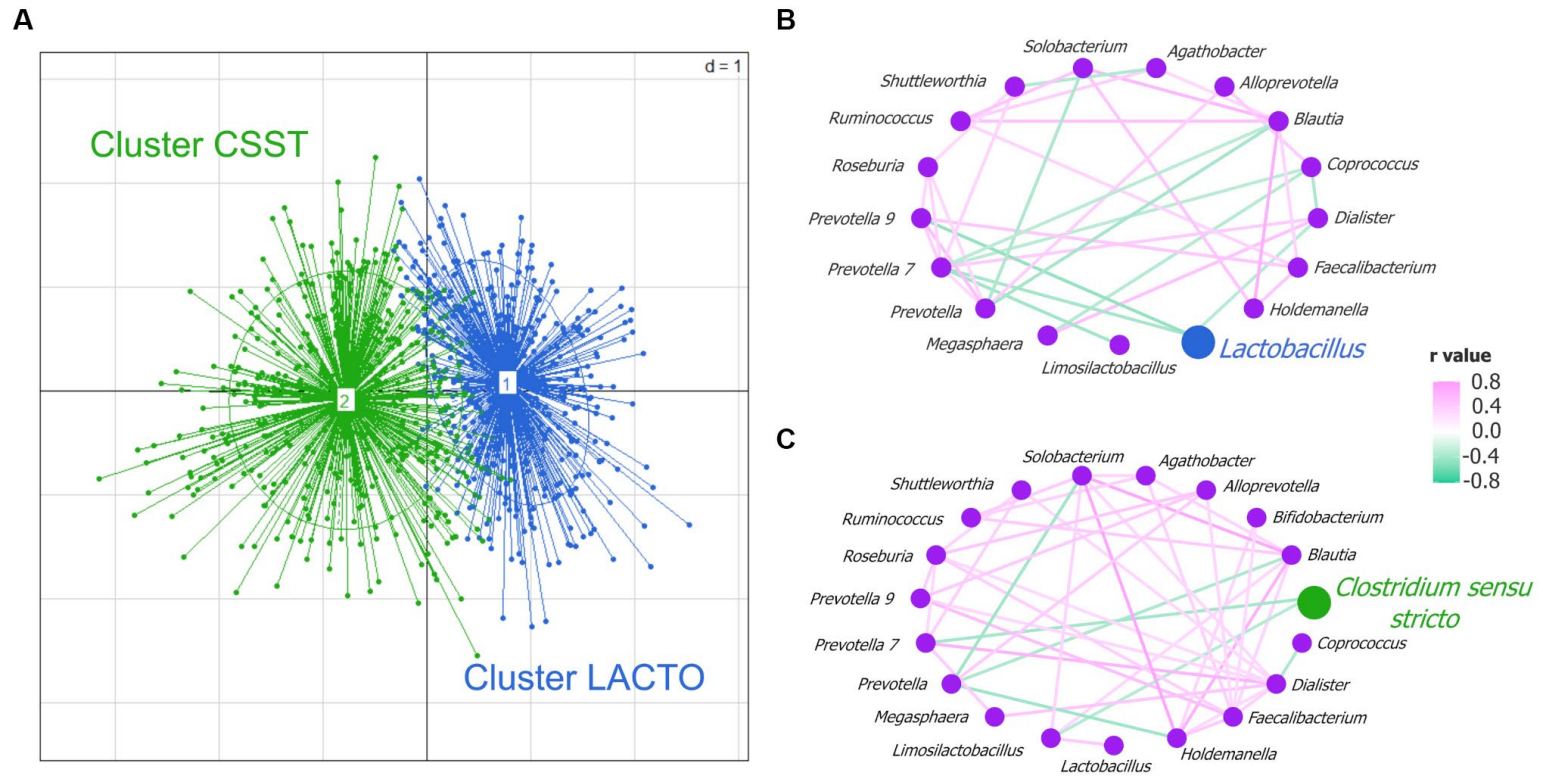
Enterotype-like clusters. **(A)** Principal coordinate analysis (PCoA) based on Jensen-Shannon divergence distance highlighting two clusters named cluster CSST in green and cluster LACTO in blue. Co-occurrence network analysis based on Spearman correlation coefficients in **(B)** cluster LACTO, and **(C)** cluster CSST.

All fecal samples (*n* = 892) were grouped into two enterotype-like clusters (Supplementary Figure S3). In accordance with the respective highest taxon weight, they were named by cluster *Lactobacillus* (LACTO) and cluster *Clostridium sensu stricto* (CSST) and were visualized in the PCoA plot (Figure 2A). To investigate potential interactions between microbial taxa, co-occurrence network analysis was performed for each enterotype-like cluster. In the network of the cluster LACTO in total 17 genera were involved (*p* < 0.0001; Figure 2B). 11 out of 34 correlations were negative. The genus *Lactobacillus* was negatively correlated with *Dialister*, *Prevotella 7* and *Prevotella 9*. The cluster CSST network contained 19 genera (*p* < 0.0001; Figure 2C). Seven out of 45 correlations were negative. Genus *Clostridium sensu stricto* was negatively correlated with *Limosilactobacillus* and *Prevotella 7*. The Shannon diversity index differed between the two clusters to a small extent (cluster LACTO: 4.49; cluster CSST: 4.54; *p* = 0.03).

The comparison of the averaged relative abundance at the genus level (averaged relative abundance >1%) revealed that almost all genera differed between cluster LACTO and cluster CSST (*p* < 0.05; Supplementary Table S5). Most genera had higher relative abundances in cluster LACTO than in cluster CSST. This was also the case for *Lactobacillus*, the most abundant genus of cluster LACTO (cluster LACTO: 21.9% and cluster CSST: 11.7%). *Streptococcus*, the most abundant genus in cluster CSST and *Clostridium sensu stricto* (19.0 and 5.6%) were both significantly less abundant in cluster LACTO (11.5 and 1.7%; *p* < 0.05). Uncl. *Lachnospiraceae* and Uncl. *Prevotellaceae* were within the unclassified bacteria, the only groups that had higher abundances in cluster LACTO than in cluster CSST (*p* < 0.05).

Pig samples that maintained their position within the same enterotype-like cluster across both sampling periods were labeled as “stable.” Those that altered their cluster affiliation were designated with the cluster name from SP 1, accompanied by the “unstable” index. Evaluation of the pigs’ affiliation to the enterotype-like cluster across both SPs resulted in the following groups: cluster LACTO (stable; *n* = 141 pigs), cluster LACTO (unstable; *n* = 142 pigs), cluster CSST (stable; *n* = 138 pigs) and cluster CSST (unstable; *n* = 25 pigs; Supplementary Figure S4A). The numbers of female and male pigs within each subgroup of the enterotype-like clusters varied and indicated an unequal sex distribution (Supplementary Figure S4B). The cluster LACTO (stable) and cluster CSST (unstable) had more male than female pigs, while the opposite occurred in the cluster CSST (stable) and cluster LACTO (unstable).

The Shannon diversity index ranged from 4.45 to 4.57 and was statistically different between the cluster LACTO (stable) and the cluster LACTO (unstable), and between both stable subgroups (*p* < 0.05).

**Figure 3:**
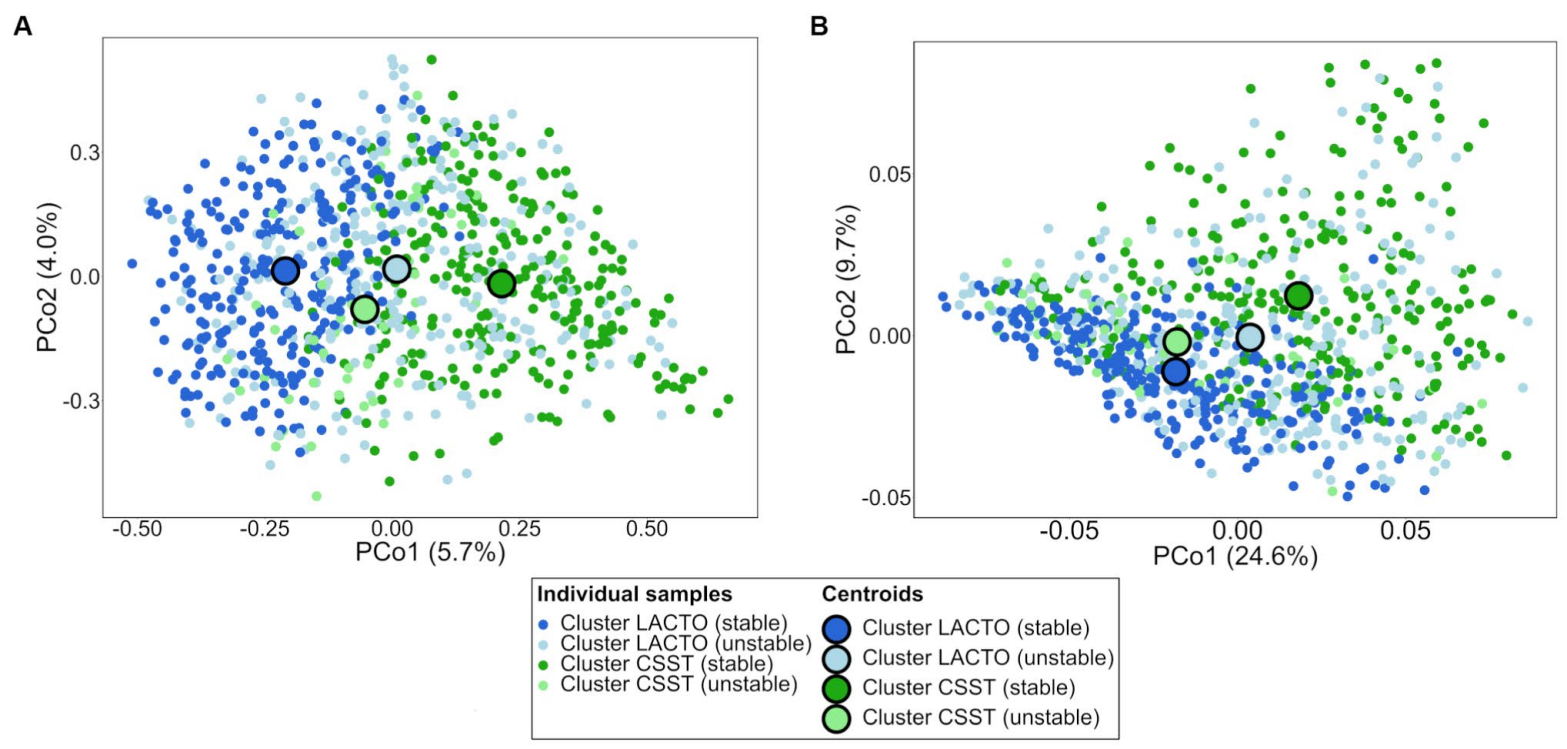
Ordination diagrams of the subgroups of the enterotype-like clusters based on Bray–Curtis dissimilarity. **(A)** Distribution of samples based on bacterial ASVs and **(B)** predicted KEGG pathways.

PCoA plot based on ASV level revealed a grouping of the samples of cluster LACTO (stable) and cluster CSST (stable), as evidenced by the spatial separation between their centroids (Figure 3A). Conversely, the proximity of centroids for the LACTO (unstable) and CSST (unstable) clusters suggested a greater similarity in their microbial compositions. The PERMANOVA confirmed the differences in microbial composition based on ASVs (*p* = 0.0001). The pairwise PERMANOVA indicated that the microbiota composition differed between all enterotype-like cluster subgroups (Supplementary Table S6).

Comparing the averaged relative abundances at the genus level revealed significant variations among the subgroups within the enterotype-like clusters (Supplementary Figure S5, Table S7). The relative abundances of all genera differed between the stable subgroups, while only specific genera, e.g., *Blautia*, *Faecalibacterium* and *Limosilactobacillus* varied within the unstable ones (*p* < 0.05). Most genera had their highest relative abundances in cluster LACTO (stable) e.g. *Dialister*, *Megasphaera* and *Prevotella 9*. *Lactobacillus*, the most abundant genus in both cluster LACTO subgroups, differed significantly across all subgroups (cluster LACTO (stable): 23% > cluster CSST (unstable): 19.3% > cluster LACTO (unstable): 16.3% > cluster CSST (stable): 10.7%). Within the subgroups of cluster CSST *Streptococcus* was the most abundant genus. The subgroup cluster CSST (stable) exhibited the highest relative abundance of this genus and those of *Clostridium sensu stricto* (*Streptococcus*: 19.7% and *Clostridium sensu stricto*: 5.5%). The lowest relative abundances of both genera were revealed in cluster LACTO (stable; *Streptococcus*: 11% and *Clostridium sensu stricto*: 1.7%). No significant differences were revealed between the unstable subgroups (*Streptococcus* both subgroups: 15%, *Clostridium sensu stricto* cluster LACTO: 4% and *Clostridium sensu stricto* cluster CSST: 2.5%).

**Figure 4:**
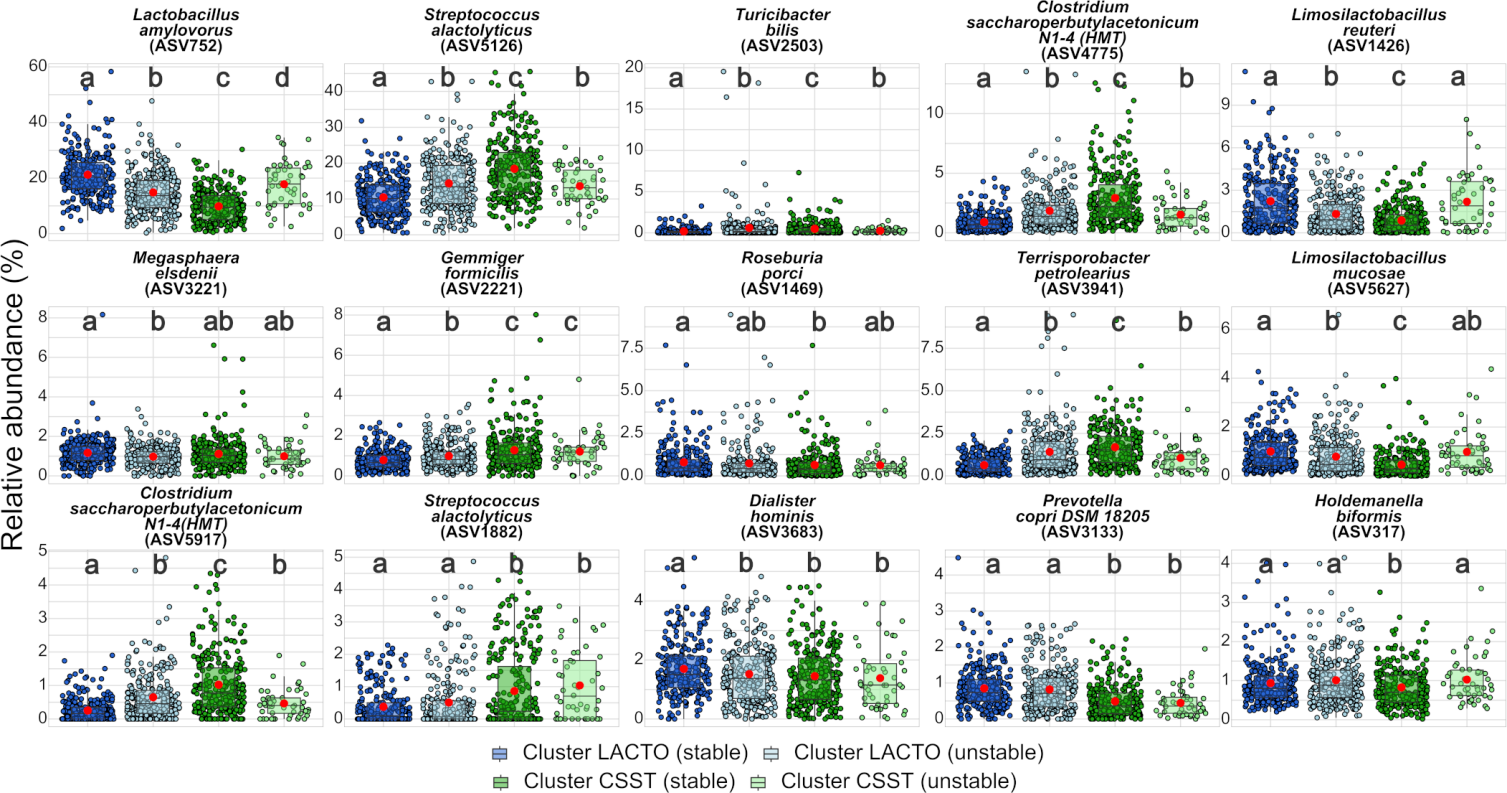
Top 15 ASVs. Comparison of the relative abundance between the subgroups of enterotype-like clusters. Red dots represent the mean within each subgroup of the enterotype-like cluster. Different letters within each ASV indicate significant differences (*p* < 0.05).

Fifteen ASVs contributed to 30% of dissimilarity between cluster LACTO and cluster CSST (Supplementary Table S8). *Lactobacillus amylovorus* (ASV752) and *Streptococcus alactolyticus* (ASV5126) contributed the most with 9.9 and 8.5%, respectively, followed by the remaining ASVs, which provided less than 2% each. All ASVs were further analyzed comparatively between the enterotype-like cluster subgroups (Figure 4; Supplementary Table S9). *L. amylovorus* (ASV752) and *S. alactolyticus* (ASV5126) were the most abundant ASVs within all subgroups. For both ASVs, the significant highest and lowest relative abundances were detected in the stable subgroups, but not in the same enterotype-like cluster. In cluster LACTO (stable) was the relative abundance of *L. amylovorus* (ASV752; 21.2%) more than twice that in cluster CSST (stable; 9.8%). Between all subgroups, differences were revealed (*p* < 0.05). For *S. alactolyticus* (ASV5126) the significant highest and lowest relative abundances were exposed in cluster CSST (stable) and in cluster LACTO (stable) with proportions of 18.4 and 10.4%, while no differences were found among the unstable subgroups (*p* > 0.05; Supplementary Table S9). *Clostridium saccharoperbutylacetonicum N1-4* (*HMT*; ASV4775), *Terrisporobacter petrolearius* (ASV3941) and *C. saccharoperbutylacetonicum N1-4* (*HMT*; ASV5917) followed the same pattern as *S. alactolyticus* (ASV5126) and had the highest and lowest relative abundances in cluster CSST (stable) and in cluster LACTO (stable), respectively. The highest and lowest relative abundances of *Limosilactobacillus reuteri* (ASV1426), *Roseburia porci* (ASV1469) and *Limosilactobacillus mucosae* (ASV5627) were detected in cluster LACTO (stable) and cluster CSST (stable). *Megasphaera elsdenii* (ASV3221), *Dialister holminis* (ASV3683) and *Prevotella copri DSM* (ASV3133) had the highest relative abundances in cluster LACTO (stable) with 1.2, 1.7 and 0.9%. The remaining low-abundant ASVs with relative abundances between 0.2 and 2.9% differed most often only marginal between the subgroups (Figure 4; Supplementary Table S9).

### 3.3 Functional prediction

The taxonomic dataset was used to predict metabolic pathways encoded in the respective taxa of the enterotype-like cluster subgroups. The distribution of the samples based on predicted KEGG pathways showed differences between the stable and unstable subgroups’ centroids indicating considerable differences in their functionality (Figure 3B). Differences in the proportional composition of KEGGs was observed among the subgroups of the enterotype-like clusters (*p* < 0.001). The pairwise comparisons revealed that the functional composition differed significantly between the subgroups of the enterotype-like clusters, except for cluster LACTO (stable) vs. cluster CSST (unstable; Supplementary Table S10). The 62 KEGG pathways that remained after filtering for “Amino acid metabolism,” “Carbohydrate metabolism,” “Lipid metabolism,” “Metabolism of other amino acids” and “Energy metabolism” had an average proportion of 23.3% of all pathways detected in the samples.

Nine out of the top 30 discriminating KEGG pathways detected by LEfSE (Supplementary Figure S6A, Table S11) belonged to “Amino acid metabolism” and “Carbohydrate metabolism.” “Lipid metabolism” and “Energy metabolism” were represented with five KEGGs each, whereas “Metabolism of other amino acids” pathways were only present with two KEGGs. Most of the revealed KEGG pathways (in total 24) were attributed to the enterotype-like cluster CSST subgroups. The subgroups of cluster LACTO were mainly discriminated by KEGGs belonging to “Carbohydrate metabolism” and “Energy metabolism” (e.g., starch and sucrose metabolism and oxidative phosphorylation). Cluster LACTO (unstable) was differentially enriched with KEGGs of the “Carbohydrate metabolism” (i.e., amino sugar and nucleotide sugar metabolism). Only cluster LACTO (stable) was further distinguished by the enrichment of one KEGG involved in “Amino acid metabolism” (cysteine and methionine metabolism). The stable subgroup within cluster CSST was mainly discriminated by KEGGs belonging to “Amino acid metabolism” (in total six KEGGs). In contrast, the unstable subgroup was distinguished by a higher number of KEGGs belonging to “Lipid metabolism” (four KEGGs). The cluster CSST (stable) showed the highest LDA score for the KEGG “Phenylalanine, tyrosine and tryptophan biosynthesis” which is also related to the “Amino acid metabolism.” Only the stable and unstable subgroup of the enterotype-like cluster CSST were characterized by the differential enrichment of KEGGs belonging to “Metabolism of other amino acids” (beta-Alanine and glutathione metabolism). The average relative abundances of the pathways described above varied among the subgroups of the enterotype-like clusters in small proportions (*p* < 0.05; Supplementary Figure S6B, Table S11). Pathways were identified up to 1.9%, such as starch and sucrose metabolism, and up to 1.6% for amino sugar and nucleotide sugar metabolism.

### 3.4 Link to metabolites, performance characteristics and blood metabolites

Fecal VFA concentrations (mmol/g), key performance indicators, and blood metabolites (all averaged across both SPs) are presented in Supplementary Table S12. Except for NUE, all data were non-normal distributed. The variance for the factor NUE was homogenous (*p* > 0.05).

**Figure 5:**
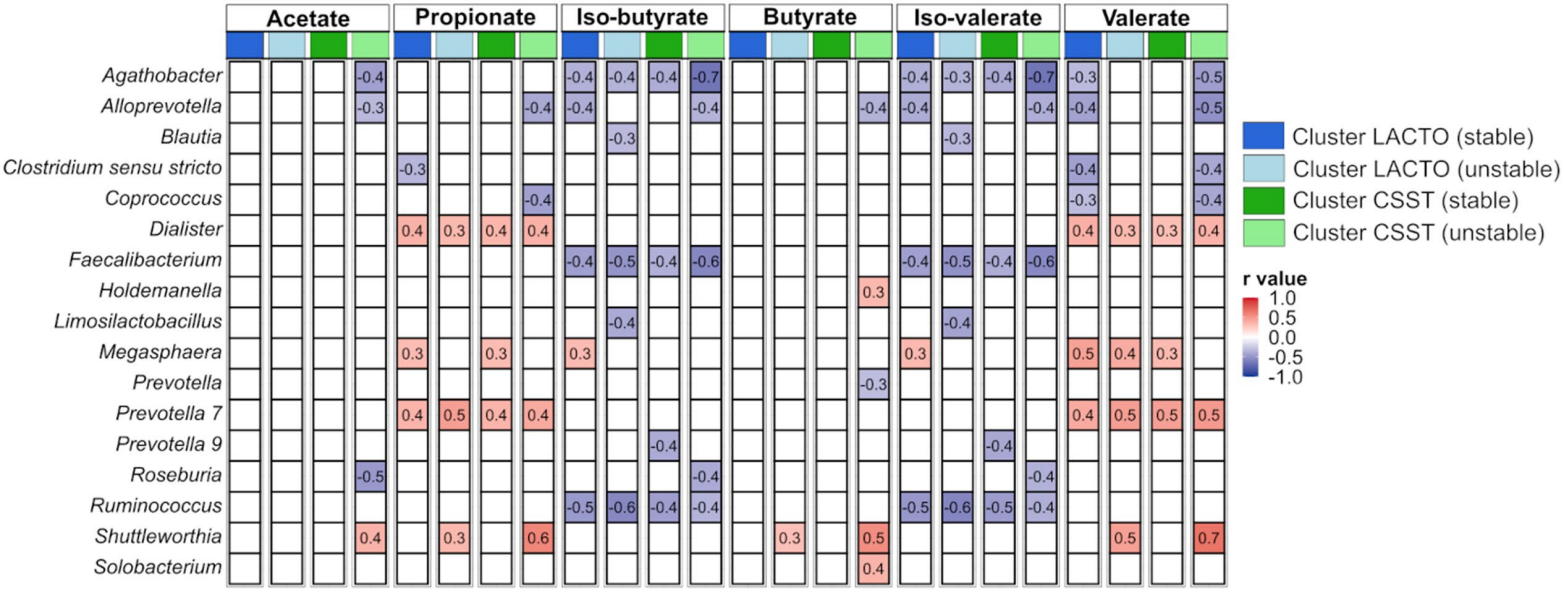
Correlations between genera and produced VFAs based on Z-scores within each subgroup of the enterotype-like clusters.

Correlations between genera (averaged relative abundance >1%, excluding unclassified genera) and VFA concentrations, averaged over both SPs, are depicted in Figure 5, and categorized by the subgroups of the enterotype-like clusters. In several cases similar correlations occurred within all subgroups, e.g., *Dialister* and *Prevotella 7* were positively correlated with propionate and valerate, and *Agathobacter*, *Faecalibacterium* and *Ruminococcus* were negatively correlated with iso-butyrate and iso-valerate. In the cluster LACTO (stable), positive correlations with branched-chain fatty acids were observed solely for *Megasphaera*. This genus also exhibited positive correlations with propionate in both stable subgroups and with valerate in both subgroups of cluster LACTO and in cluster CSST (stable). *Shuttleworthia* was only positively correlated with acetate, propionate, butyrate and valerate in the stable subgroups.

**Figure 6:**
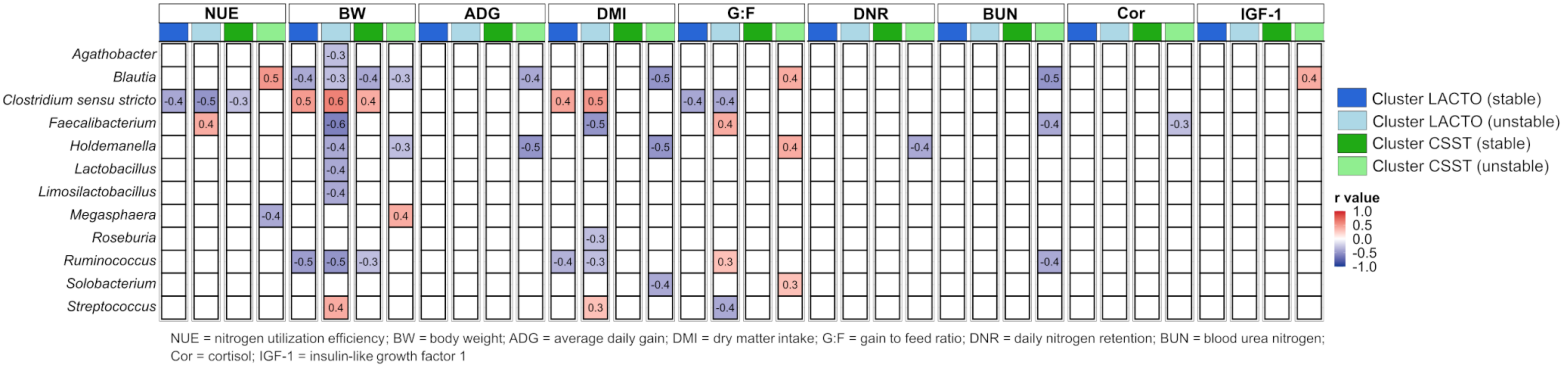
Correlations between genera and performance characteristics and blood metabolites based on Z-scores within each subgroup of the enterotype-like clusters.

Correlations between genera (averaged relative abundance >1%, excluding unclassified genera) and performance data and blood metabolites across each subgroup of the enterotype-like cluster are depicted in Figure 6. Generally, more correlations emerged in the unstable subgroups than in the stable ones. BW showed the most correlations with genera among all subgroups. Negative correlations were found with *Ruminococcus* in both subgroups of the cluster LACTO, with *Blautia* in all subgroups, and positive correlation with *Clostridium sensu stricto* in both stable subgroups and the cluster LACTO (unstable). Regarding NUE, negative associations with *Clostridium sensu stricto* in both stable subgroups and in the cluster LACTO (unstable), positive correlations within the unstable subgroups (*Blautia*: cluster CSST and *Faecalibacterium*: cluster LACTO), and negative with *Megasphaera* in cluster CSST (unstable) were observed. A few correlations were identified for ADG, DMI, G:F and DNR. In cluster CSST (unstable), blood metabolites correlated only with specific genera. *Blautia*, *Faecalibacterium* and *Ruminococcus* negatively correlated with BUN. *Faecalibacterium* showed a negative correlation with Cor, and a positive correlation was observed between IGF-1 and *Blautia*. No correlations of blood metabolites were calculated for the other enterotype-like clusters.

The calculated Z-scores for VFA concentrations (Supplementary Figures S7A–F) revealed significant variations (*p* < 0.05) among subgroups within the enterotype-like cluster (Supplementary Table S13). However, these differences were marginal in magnitude. The cluster CSST (stable) exhibited the highest Z-scores for all VFAs, with acetate (0.1) at a concentration of 69.4 mmol/kg, the only VFA showing no significant differences among the subgroups (*p* > 0.05). Propionate and valerate followed the same pattern within the subgroups: The highest Z-scores (0.2) represented concentrations of 36.2 and 5.4 mmol/kg that differed from those in the unstable subgroups (cluster LACTO: 33.4 mmol/kg and 4.6 mmol/kg, and cluster CSST: 32.2 mmol/kg and 4.5 mmol/kg). Iso-butyrate and iso-valerate showed the highest Z-scores (0.2 and 0.4), corresponding to concentrations of 3.8 and 4.6 mmol/kg, respectively. The lowest Z-scores were detected in cluster LACTO (stable; −0.2 and − 0.3) reflected concentrations of 1.9 and 2.4 mmol/kg (p < 0.05). The highest Z-score for butyrate (0.2) which expressed a concentration of 21.4 mmol/kg differed from the other subgroups (p < 0.05).

**Figure 7:**
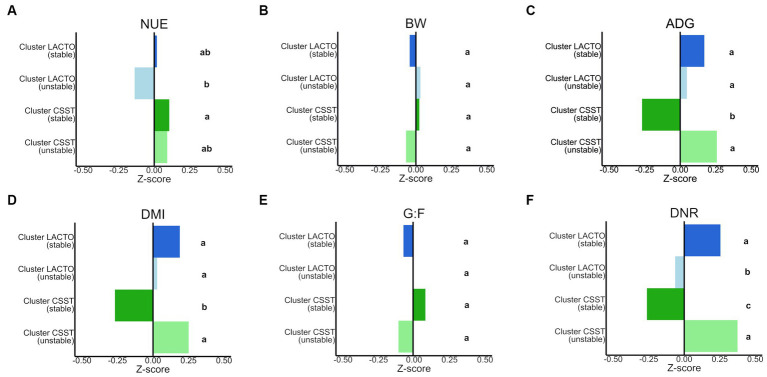
Comparison of the Z-scores of **(A)** NUE (nitrogen utilization efficiency), **(B)** BW (body weight), **(C)** ADG (average daily gain), **(D)** DMI (dry matter intake), **(E)** G:F (gain to feed ratio), **(F)** DNR (daily nitrogen retention) between subgroups of the enterotype-like clusters. Different letters indicate significant differences in the row data (*p* < 0.05).

Z-scores (Figure 7; Supplementary Figures S7G–I) determined for performance characteristics and blood metabolites varied among enterotype-like cluster subgroups (*p* < 0.05; Supplementary Table S13). The highest and lowest Z-scores for NUE were detected in cluster CSST (stable; 0.1) and cluster LACTO (unstable; −0.1) that, indicated values of 46.2 and 47%, providing the only differences between the subgroups in terms of NUE (*p* < 0.05; Supplementary Table S14). For ADG, DMI and DNR similar patterns were revealed within the subgroups of enterotype-like clusters: Cluster LACTO (stable) and cluster CSST (unstable) showed the highest Z-scores, while cluster CSST (stable) had the lowest ones (Supplementary Table S13). No differences were detected between subgroups for BW, and G:F (*p* > 0.05). Within the blood metabolites the concentrations of Cor and IGF-1 differed between the stable subgroups (*p* < 0.05).

## 4 Discussion

### 4.1 Interplay between sire, sex, SP, NUE and the fecal microbial composition

Consistent with prior research, our findings indicate minimal impact of sex on the fecal microbiota in pigs (Han et al., 2018; Wang et al., 2022). Han et al. (2018) described that fecal samples collected at five different time points could be categorized into three stages: early (10 and 21 days of age), mid (63 days of age), and late-stage (93 and 147 days of age). Male pigs were castrated before they reached the age of seven days, and although there was a slight separation between female and male samples in the early group, this differentiation disappeared in the mid and late-stage groups. The authors postulated that the sex effect on the fecal microbial composition diminishes post-castration and disappears during the post-weaning growing process. The first sampling time point of the late-stage group aligns with the two SPs in our study during the 13 and 16th weeks of life. Thus, the low impact of sex in the present study could be attributed to the similar age of the animals and the castration.

Contrary to other studies, the time of sample collection (SPs) had only a minor contribution to microbiota variation (Han et al., 2018; Wang et al., 2022). Both SPs were only 21 days apart. By this stage, the pigs had long weathered the weaning phase, known for inducing abrupt social, dietary, and environmental changes that alter the fecal microbiota (Campbell et al., 2013). Various studies have shown that the microbiota in the feces of pigs changes with increasing age (Zhao et al., 2015; Han et al., 2018). The age at which the fecal microbiota stabilizes varies across studies, generally from 3 to 6 months (Zhao et al., 2015; Han et al., 2018). Dietary changes are often considered the primary cause of gut microbiota alteration (Muegge et al., 2011; Tilocca et al., 2017; Wang et al., 2019). In pig production, the dietary composition is determined by the growth stage of the animal (Han et al., 2018). In the present study, the transition to the grower feed, which occurred in the 14th week of life, with a slight change in the proportion of ingredients, coupled with the close timing of our sample collection, might explain why the timing of the sample collection had only a minor impact.

Previous studies have established that the pig’s genetic background influences the fecal microbiota, although the extent of its influence was not reported (Pajarillo et al., 2014; Xiao et al., 2017; Wang et al., 2022). Unlike these studies that compared microbiota across different breeds, the present study examined genetic variations within a single breed, focusing on the offspring’s sire as the source of genetic difference. The large R^2^ value for the factor sire, suggests that the sire’s genetics, used for selective breeding, had a considerable influence on the fecal microbial composition of their offspring. Berghaus et al. (2023) revealed that the average NUE in SP 1 was higher than in SP 2, and found variations in the NUE between the offspring from different sires (Berghaus, 2022). Differences in the fecal microbiota composition related to the predisposition for NUE (NUE_High_ and NUE_Low_) were observed only in SP 1. At the genus level, *Streptococcus* was a significantly differentially abundant genus between NUE_High_ and NUE_Low_ group (*p* < 0.05; NUE_High_: 16.45%; NUE_Low_: 12.92%). The LinDA approach is valuable for identifying potential genera for further biological assessment (Zhou et al., 2022). Therefore, an increased relative abundance of *Streptococcus* might be considered as a potential biomarker of increased NUE at SP 1. Various *Streptococcus* species such as *S. infantarius* and *S. sali*var*ius* are commonly used in animal nutrition as probiotics (Yirga, 2015). Probiotics can improve nutrient utilization and growth performance through an improvement of gut health (better nutrient absorption by the epithelial membranes) and nutrient digestion (Liao and Nyachoti, 2017). Given that the investigated sample material was feces, it remains unclear if the NUE_High_ pigs had a relatively higher colonization of *Streptococcus* already in the small intestine than the NUE_Low_ pigs, potentially leading to beneficial health effects. A higher N utilization in the small intestine of the NUE_High_ pigs could also turn N a limiting factor for the microbiota in the subsequent gut compartments. A cultivation trial showed that *Streptococcus* species can adapt to N-limitation, when excess of glucose is present (Carlsson and Griffith, 1974). On the other hand, species like *S. bovis* produce dipeptidyl peptidase and dipeptidase, suggesting their potential importance for protein digestion and AA absorption in the GIT of pigs (Wallace, 1996; Dai et al., 2011). However, no studies have yet explored the relationship between the fecal microbiota and the NUE in pigs with a defined family structure. As the pig selection only included those belonging to the 25 and 75% quantiles, a significant number of pigs (223 in total) and microbiota data were not considered. For some sires, the offspring was either entirely absent or unevenly represented in the NUE_High_ and NUE_Low_ groups. This observation suggests that a genetic component might also influence the NUE predisposition at SP 1 regarding the fecal microbiota. Genomic analysis with the same data set showed a heritability of NUE (Schmid et al., 2024).

### 4.2 Exploration of the enterotype-like clusters in the fecal microbiota

Enterotyping, a method first described in human microbiome research (Arumugam et al., 2011) and later applied to several pig studies, simplifies the complexity of microbiota data by creating distinct clusters within the gut microbiota (Costea et al., 2018). In pigs, enterotypes have been associated with age (Lu et al., 2018; Le Sciellour et al., 2019), sex (Le Sciellour et al., 2019), diets (before and after weaning; Ke et al., 2019), and breeds (Ma et al., 2022). In the present study, two enterotypes were identified in 892 fecal samples from 446 pigs. Contrary to most pig studies, the clusters were identified across the entire dataset, regardless of sampling time point (Lu et al., 2018; Ke et al., 2019; Le Sciellour et al., 2019). The main reason was the very low R^2^ value for the factor SP as previously discussed. With the exception of the study by Le Sciellour et al. (2019) (*n* = 160 pigs), these studies also investigated large cohorts of pigs. Lu et al. (2018) studied 1,039 pigs within each time point, while Ke et al. (2019) examined 953 pigs. Interestingly, the identified enterotype-like clusters of the present study were more continuous than discrete, deviating from the traditional definition of enterotypes. This aligns with the findings of a human microbiota meta-study conducted by Koren et al. (2013), which proposed that bacterial abundances across most body sites follow a gradient, thus leading to enterotypes with blurred boundaries (i.e., indiscrete enterotypes). In very few cases, they found discrete community types (e.g., in the vagina). Most studies investigating the fecal pig microbiome at different time points identified two clusters at each time point (Ramayo-Caldas et al., 2016; Lu et al., 2018; Ke et al., 2019). Le Sciellour et al. (2019) identified two clusters in fecal samples (day 52, 99, 140, and 154) and a third one on day 119. Xu et al. (2021) explored the fecal microbiota of Jinhua pigs at 105 days and uncovered three clusters. Enterotypes can be identified by the varying levels of key genera driving the distinctions between clusters (Arumugam et al., 2011). In the present study, the major drivers for the two enterotype-like clusters were *Lactobacillus* and *Clostridium sensu stricto*. However, the identified driver genera can considerably vary among different studies (Mach et al., 2015; Lu et al., 2018; Ke et al., 2019; Xu et al., 2021). Ke et al. (2019) identified *p-75-a5* and *Fusobacterium* as the main drivers at day 25 and *Prevotella* and *Treponema* for days 80, 120, and 240. The authors attributed the occurrence of the drivers to the feeding before and after weaning. Like our study, Xu et al. (2021) revealed *Lactobacillus* and *Clostridium sensu stricto 1* as the main drivers for the first two enterotypes. However, they detected a third enterotype driven by *Bacteroides*. Mach et al. (2015) identified *Prevotella* and *Ruminococcus* as major drivers. This wide variation highlights the complexity of microbial ecosystems and the importance of considering the study design, including sample processing and data analysis, when interpreting results. Microbial community exhibits considerable variation influenced by factors such as the ecological relationship between the microbial colonizers, whether opportunistic or symbiotic (Faust et al., 2012). Co-occurrence network analysis has been done to delve into potential interactions among microbial taxa to provide a more comprehensive understanding of the microbial community structure (Barberán et al., 2012). Within the network of the cluster LACTO, the genus *Lactobacillus* was negatively correlated to *Dialister*, *Prevotella 7* and *Prevotella 9*. In the cluster CSST network, the genus *Clostridium sensu stricto* correlated negatively with *Limosilactobacillus* and *Prevotella 7*. Our findings diverge from the study of Arumugam et al. (2011), as in our study, the identified enterotype-like clusters were not group-driven, because the major drivers showed no positive association with other members. The Shannon diversity index differed slightly between the two enterotype-like clusters (cluster LACTO: 4.49; cluster CSST: 4.54; *p* < 0.05). At this point, it can already be stated that the differences between the subgroups were also marginal (range between 4.45 and 4.57), but still statistically different between the two cluster LACTO subgroups and both stable subgroups (*p* < 0.05). Le Sciellour et al. (2019) reported a significant difference in the Shannon diversity index only at day 52. The enterotype dominated by *Prevotella–Sarcina* had a lower Shannon diversity index than the one dominated by *Lactobacillus* (6.60 vs. 6.76). In contrast, Lu et al. (2018) reported no significant differences between the two enterotypes identified at day 105 (4.50 and 4.47, respectively). Furthermore, Han et al. (2018) proposed that the diversity of the microbial community is age-related in pigs and demonstrated a trend toward less diverse fecal microbiota as the pigs aged. This might explain the lower Shannon diversity index in the current study compared to the results of Le Sciellour et al. (2019). However, the association between the Shannon diversity index and age has not been fully clarified. Gaire et al. (2022), postulated that the microbial community in growing pigs becomes more diverse and presumably more stable, with increasing age. Increased diversity is associated with improved robustness of the gut microbiota (Larsen and Claassen, 2018). It’s strongly associated with fat thickness and ADG (Lu et al., 2018). The current study could not detect a connection between ADG and diversity.

#### 4.2.1 Subgrouping of the enterotype-like clusters

Examining sample stability from SP 1 to SP 2, only 141 out of 446 pigs consistently belonged to the LACTO cluster, while 138 remained in the CSST cluster. Nearly one-third of the pigs changed their affiliation to a specific enterotype-like cluster between SP 1 and SP 2. Other studies observed that a pig’s affiliation with an enterotype changes over time, either post-weaning (Mach et al., 2015), between weaning and finishing (Lu et al., 2018) or during the finishing stage (Le Sciellour et al., 2019). The factors influencing whether a pig stays within or transition between enterotype-like clusters over time are of particular interest. In the current study, the persistence within an enterotype-like cluster appeared to be sex-dependent. Interestingly, the cluster LACTO (stable) had more males than females, while the contrary was observed in the cluster CSST (stable). Le Sciellour et al. (2019), also observed a sex-dependent association with enterotype-like clusters but solely at day 99. Similar to the present study, less females were clustered in the *Lactobacillus* enterotype than in the *Clostridium sensu stricto-Turicibacter* enterotype. However, the males, that were castrated as in the present study, were evenly distributed in both enterotypes. Mach et al. (2015) identified lactation-associated genera as potential biomarkers, indicating whether pigs would shift or remain stable in their enterotype-like cluster after weaning. A lower abundance of *Clostridia* and a higher abundance of *Lactobacillus* in 14-day-old suckling pigs indicated the shift in the *Prevotella* cluster after weaning. The abundance of *Lactobacillus fermentum* was twice as high in the feces of pigs that remained consistently in the *Prevotella* cluster (Mach et al., 2015). Lu et al. (2018) suggested that the pig’s genetic background affected the partitioning into enterotype-like cluster (Lu et al., 2018). Whether a similar genetic influence affected the stability of cluster affiliation in the current study remains to be determined. Previously, it was shown the effect of genetics on gut microbiota (Camarinha-Silva et al., 2017). In the present study the significant impact of genetics on the composition of the microbial community in the feces of the offspring from different sires (evidenced by the high R^2^ value) indicated that this factor appears promising in the context of cluster affiliation.

The subgroups of the enterotype-like clusters were characterized by different microbiota community structures (genus and ASV level), predicted functional architecture (KEGGs) and produced metabolites (VFAs). These variations were observed, although all pigs were kept under standardized conditions, including administering high- grain-based diets in both phases, as is common in commercial pig farming (Zijlstra et al., 2010). Dietary composition is critical in shaping the gut microbiota (Tilocca et al., 2017). Carbohydrates and proteins that resist enzymatic digestion in the proximal intestine undergo bacterial fermentation, producing VFAs (Krautkramer et al., 2021). Branched-chain fatty acids are produced through proteolytic fermentation (Mortensen and Clausen, 1996). This implies that a higher fecal concentration of these VFAs might indicate a larger proportion of proteolytically active bacteria in the proximal gut (Krautkramer et al., 2021).

Across both SPs, the cluster CSST (stable) pigs had the highest concentration of all identified VFAs (highest Z-scores) in the feces. These were significantly different in several cases from those measured in the other subgroups, except acetate. The group of proteolytic active bacteria is diverse and includes saccharolytic bacteria up to obligate AA fermenters (Davila et al., 2013). Representatives in the large intestine microbiota community that exhibit these characteristics are, for example, *Streptococcus* and *Lactobacillus* (Davila et al., 2013). In the present study, both genera had high proportions in the subgroups of the enterotype-like clusters. However, the abundance of *Streptococcus* followed the same pattern as the Z-scores for the branched-chain fatty acids. This suggests that species of this genus are more likely to be proteolytically active members than species of *Lactobacillus*. The effect of *Streptococcus* on the host intestine is less studied than *Lactobacillus* (Lan et al., 2023). *Clostridium sensu stricto*, the genus with the second-highest taxon weight in the cluster analysis, is one of the main genus detected in the feces of pigs after weaning (Chen et al., 2017) and its relative abundance increases with age (Gaire et al., 2022). Currently, no study has shown proteolytic capabilities in this genus.

The present study explored *L. amylovorus* (ASV752) and *S. alactolyticus* (ASV5126) as the ASVs that contributed most to the dissimilarity of clusters LACTO and CSST. The evaluation of the proteolytic ability of LAB group members was of central interest in previous studies (Liu et al., 2010; Kieliszek et al., 2021; Christensen et al., 2023). However, it was revealed that the proteolytic ability can vary widely (Liu et al., 2010; Christensen et al., 2023). Although all members are equipped with a proteolytic system consisting of proteinases, transport proteins and peptidases, there are inter-species differences (Kieliszek et al., 2021). The proteolytic system genes are distributed unevenly among different strains (Liu et al., 2010). The knowledge about the distribution that is indispensable to predicting the proteolytic potential of different strains (Liu et al., 2010) is still lacking and needs future research. *L. reuteri* (ASV1426) and *L. mucosae* (ASV5627) had their highest and lowest relative abundances in cluster LACTO (stable) and cluster CSST (stable). *Limosilactobacillus reuteri* and *Lactobacillus amylovorus* are colonizers of pig intestine and synthesize bacteriocins, which can inhibit the growth of other intestinal microorganisms (Hammes and Hertel, 2006). The administration of *Limosilactobacillus mucosae* LM1 in pig diets as a probiotic (moderate-high doses) led to an increase of the apparent total tract digestibility of N and an improvement of the immune response by reducing serum pro-inflammatory cytokines (interleukin-1β and tumor necrosis factor-alpha) and increasing anti-inflammatory cytokines (interleukin-10; Zhang et al., 2022). In the current study, despite the higher fecal abundance of *Limosilactobacillus mucosae* in the cluster LACTO (stable), no increase in N utilization were revealed (Z-score for NUE close to the average). In contrast, *Clostridium saccharoperbutylacetonicum N1-4* (*HMT*; ASV4775), *Terrisporobacter petrolearius* (ASV3941) and *Clostridium saccharoperbutylacetonicum N1-4* (*HMT*; ASV5917) had the highest and lowest relative abundances in cluster CSST (stable) and in cluster LACTO (stable). *C. saccharoperbutylacetonicum* is a cellulolytic bacteria (Tran et al., 2018) that plays a key role in the breakdown of cellulose, which is indigestible by the host, and contributes to its health by producing VFAs and maintaining the gut microbiota (Froidurot and Julliand, 2022). In the present study, no correlations were detected between *Lachnoclostridium*, the corresponding genus of *C. saccharoperbutylacetonicum* and the VFAs. *Turicibacter bilis* (ASV2503) had the highest abundance in the cluster LACTO (unstable). Although *T. bilis* is known to produce acetate, butyrate and lactate (Maki and Looft, 2022), this study found no positive association between the genus *Turicibacter* and acetate or butyrate. *Prevotella copri DSM* (ASV3133) and *Megasphaera elsdenii* (ASV3221) had the highest relative abundances in cluster LACTO (stable). Chen et al. (2021) revealed that *P. copri* was associated with fat storage in pigs and its abundance was positively related to increased concentrations of serum metabolites associated with obesity, e.g., lipopolysaccharides or branched-chain AA. On the other hand, the host intestinal barrier was more permeable, and the chronic inflammatory response increased. *M. elsdenii* can transform lactate to butyrate, which is physiologically vital for the hindgut mucosa (Hashizume et al., 2003). *M. elsdenii* synthesize highly active dipeptidyl peptidase and dipeptidase, indicating that these bacteria might be important for protein digestion and AA absorption in the GIT (Wallace, 1996; Dai et al., 2011). The present study found only positive correlations for *Megasphaera* and the branched-chain fatty acids within cluster LACTO (stable). However, the lowest concentrations of iso-butyrate and iso-valerate were detected in this subgroup. A possible reason for the exclusive position of the cluster CSST (stable) regarding the VFA concentrations over both SP could be the distribution of the differentially abundant KEGGs (detected by LEfSe). Most of the predicted KEGGs belonging to the AA metabolism were assigned to this subgroup. *Streptococcus*, the most abundant genus in this subgroup, is known to synthesize AA *de novo* (Willenborg and Goethe, 2016) that can be further utilized.

In the present study several negative correlations were detected between the genera, e.g., *Agathobacter*, *Faecalibacterium* and *Ruminococcus* and the branched-chain fatty acids within all subgroups. During the protein fermentation process, potentially toxic by-products such as ammonia are formed (Mortensen and Clausen, 1996; Bikker et al., 2007). A higher protein fermentation rate increases the branched-chain fatty acid concentrations and led to the accumulation of potentially harmful by-products (Birkett et al., 1996) in the feces. This accumulation may, in turn, inhibit the growth of other genera such as *Agathobacter*, *Faecalibacterium* and *Ruminococcus*.

Positive correlations were observed between the genera *Dialister*, *Megasphaera*, *Prevotella 7* and *Shuttleworthia*, and the remaining VFAs. The correlations between *Dialister* and propionate within each subgroup align with the findings of Sakamoto et al. (2020) who detected succinate utilization ability in a *Dialister hominis* ssp. (also part of the top 15 ASVs in this study), and found that it could decarboxylate succinate to propionate (Sakamoto et al., 2020). Positive correlations were found between *Megasphaera* and propionate (stable subgroups of both enterotype-like clusters) and valerate (in all subgroups of enterotype-like clusters except for cluster CSST (unstable)). Species of *Megasphaera, e.g., Megasphaera elsdenii* can utilize lactate and convert D-lactate to acetate, propionate, butyrate and valerate (Counotte et al., 1981; Hashizume et al., 2003; Jiang et al., 2016). The saccharolytic bacteria *Prevotella 7* showed positive associations with propionate and valerate among each subgroup of the enterotype-like clusters. In both unstable subgroups, *Shuttleworthia* was positive associated with butyrate concentration. *Shuttleworthia* is a saccharolytic bacteria that produces acetate, butyrate and lactate as end products from glucose fermentation (Downes et al., 2002). In contrast to this study, Xu et al. (2021) revealed a strong positive correlation between *Blautia* and *Faecalibacterium* with fecal butyrate concentration.

Previous research has proven that enterotype-like clusters are generally associated with performance traits (Mach et al., 2015; Ramayo-Caldas et al., 2016; Lu et al., 2018; Yang et al., 2018; Le Sciellour et al., 2019) and tend to be related to feed efficiency (Yang et al., 2017). For example, 60-day-old pigs belonging to the *Prevotella* and *Mitsuokella* enterotype had a BW that was 850 g heavier and an ADG that was 17.9 g higher compared to pigs of the *Ruminococcus* and *Treponema* enterotype (Ramayo-Caldas et al., 2016). The pigs belonging to cluster CSST (stable) had a 0.8% increase in NUE compared to pigs in cluster LACTO (unstable). On the other hand, the same pigs had the worst performances in terms of ADG, DMI and DNR compared to the other enterotype-like clusters. G:F is a commonly used measure of feed efficiency (Lu et al., 2017). Pigs with comparable G:F can differ greatly in their ADG and DMI (Smith et al., 2010). Variations in DMI could be triggers for increased or decreased ADG. This is consistent with previous research by Yang et al. (2018), which found a positive correlation between average daily feed intake and ADG. The altered DMI despite consistent conditions such as *ad libitum* feeding and water, may be influenced by the gut microbiota of the pigs. They suggested that pigs with similar gut microbiota may exhibit similar appetites (Yang et al., 2018). Some gut bacteria might suppress feed intake by producing VFAs and lactic acid, and regulating the metabolism of AAs (Yang et al., 2018). The differences between the subgroups for the VFAs, KEGGs and performance characteristics were rather small. These small differences are most likely due to the fact that pigs from a population selected for growth are less diverse than pigs from other studies (Lu et al., 2018). The strict standardized experimental procedure minimized possible stressors that could also cause variations in performance (Wellock et al., 2004).

This study, conducted on crossbreed fattening pigs with a defined family structure, explored the correlation between the fecal microbiota, their metabolites, and NUE. *Streptococcus* emerged as a potential biomarker when comparing pigs with high and low NUE in SP 1. Cluster analysis revealed that cluster CSST (stable) showed the highest NUE, despite containing low-performing pigs in terms of ADG, DMI and DNR. This work offers a critical understanding of the microbiome’s impact on NUE, establishing a foundation for subsequent strategies to improve sustainability in pig production.

## Data availability statement

The datasets presented in this study can be found in online repositories. The names of the repository/repositories and accession number(s) can be found at: https://www.ebi.ac.uk/ena, PRJEB70427.

## Ethics statement

The animal study was approved by Regierungspräsidium Tübingen, Germany. The study was conducted in accordance with the local legislation and institutional requirements.

## Author contributions

NS: Methodology, Writing – original draft, Writing – review & editing, Data curation, Formal analysis, Investigation. JS: Writing – review & editing, Conceptualization, Supervision. JB: Conceptualization, Writing – review & editing. MR: Conceptualization, Writing – review & editing. AC-S: Conceptualization, Writing – review & editing, Funding acquisition, Methodology, Supervision, Writing – original draft.
