# Supplementary material for: Microbial signatures and enterotype clusters in fattening pigs: implications for nitrogen utilization efficiency
Source: Front Microbiol. 2024 Apr 10;15:1354537. doi: 10.3389/fmicb.2024.1354537 (PMC11040106; doi:10.3389/fmicb.2024.1354537)
Supplement: Supplementary file 2 [file Data_Sheet_1.PDF]

Microbial signatures and enterotype clusters in fattening pigs: implications for nitrogen utilization efficiency

Naomi Sarpong[1,2], Jana Seifert[1,2], Jörn Bennewitz[1,2,] Markus Rodehutscord[1,2], Amélia Camarinha-Silva[1,2*]

[1]1Institute of Animal Science, University of Hohenheim, Stuttgart, Germany

[2]HoLMiR - Hohenheim Center for Livestock Microbiome Research, University of Hohenheim, Stuttgart, Germany

* Correspondence:
Amélia Camarinha-Silva
amelia.silva@uni-hohenheim.de

Supplementary Material

1 Supplementary Figures and Tables

1.1 Supplementary Figures

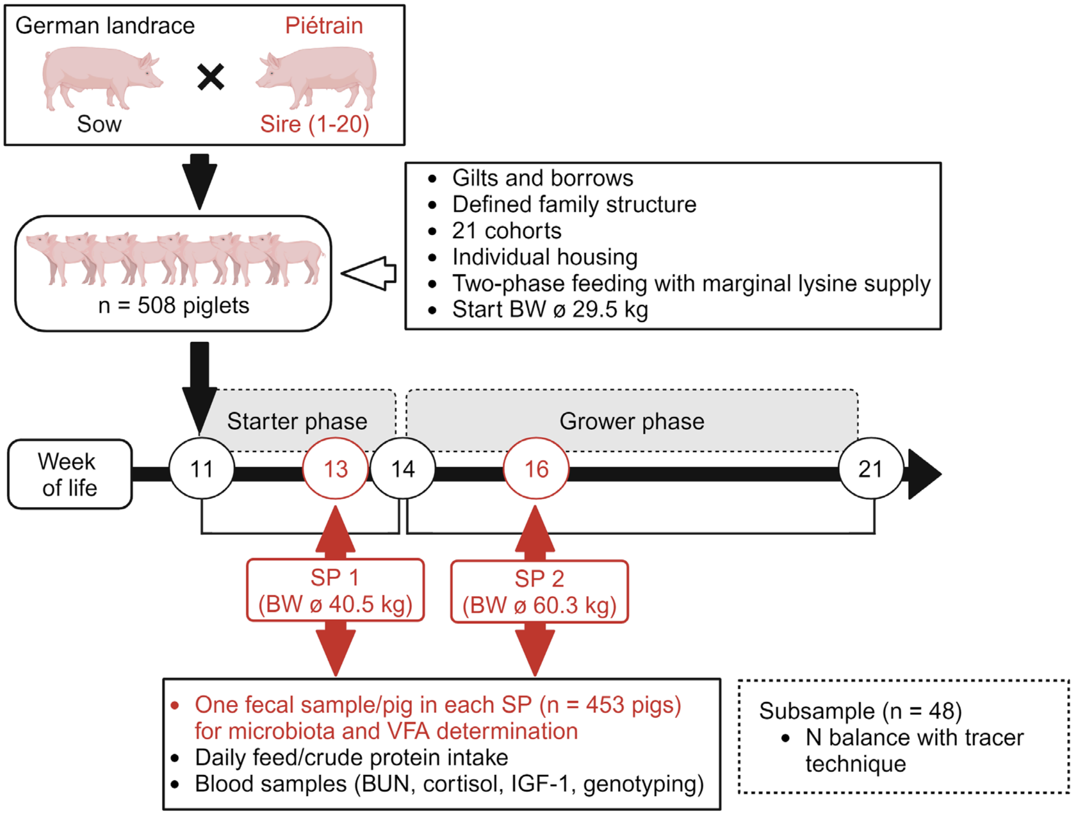

Figure S1. Experimental design.

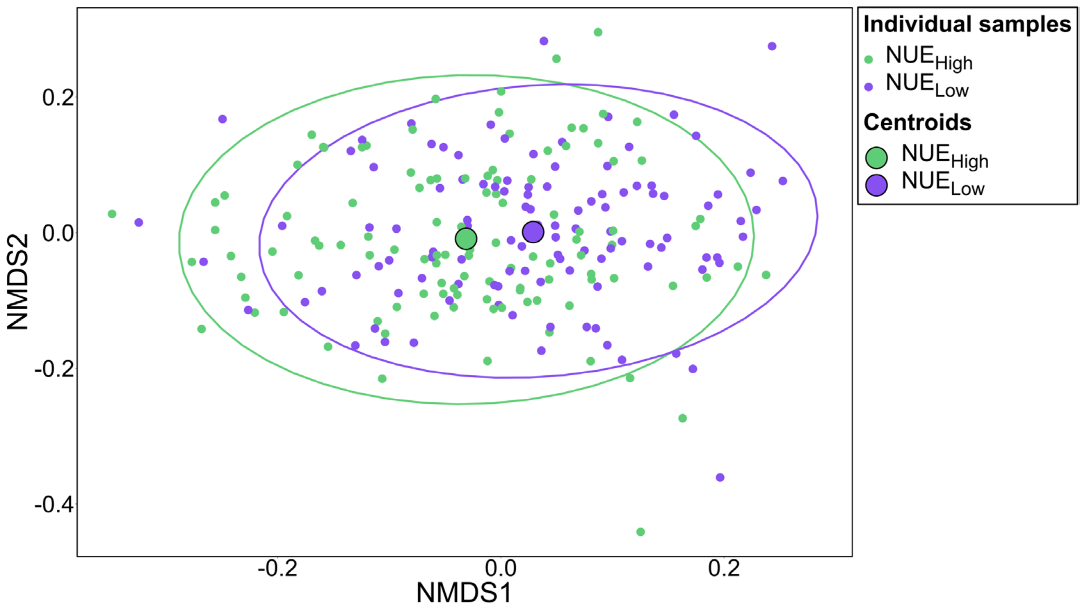

Figure S2. NMDS of SP 1. Distribution of the fecal samples of pigs belonging to NUE$_{High}$ and NUE$_{Low}$ group.

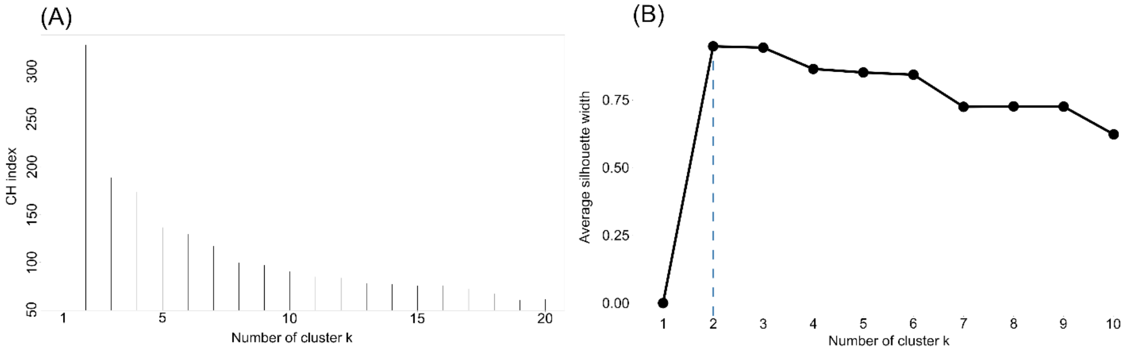

Figure S3. Optimal number of clusters. Identification by using (A) Calinski-Harabasz (CH) Index and (B) average silhouette width.

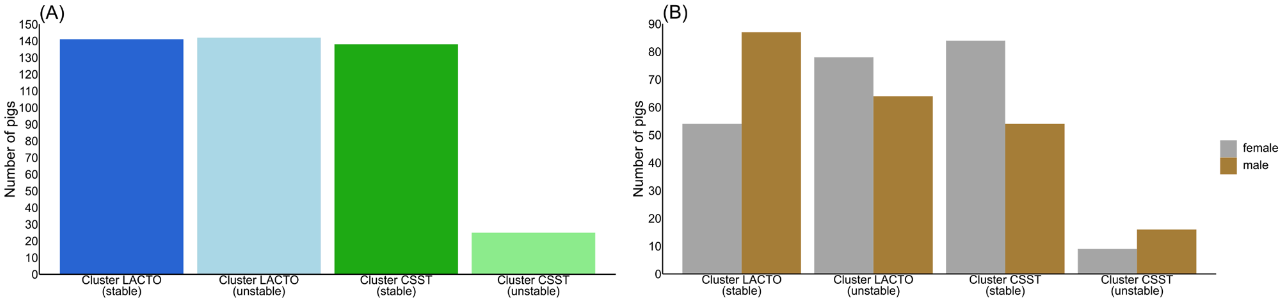

Figure S4. Number of pigs (n = 446). Assigned to each subgroup of enterotype-like cluster (A) and divided by sex within each subgroup (B).

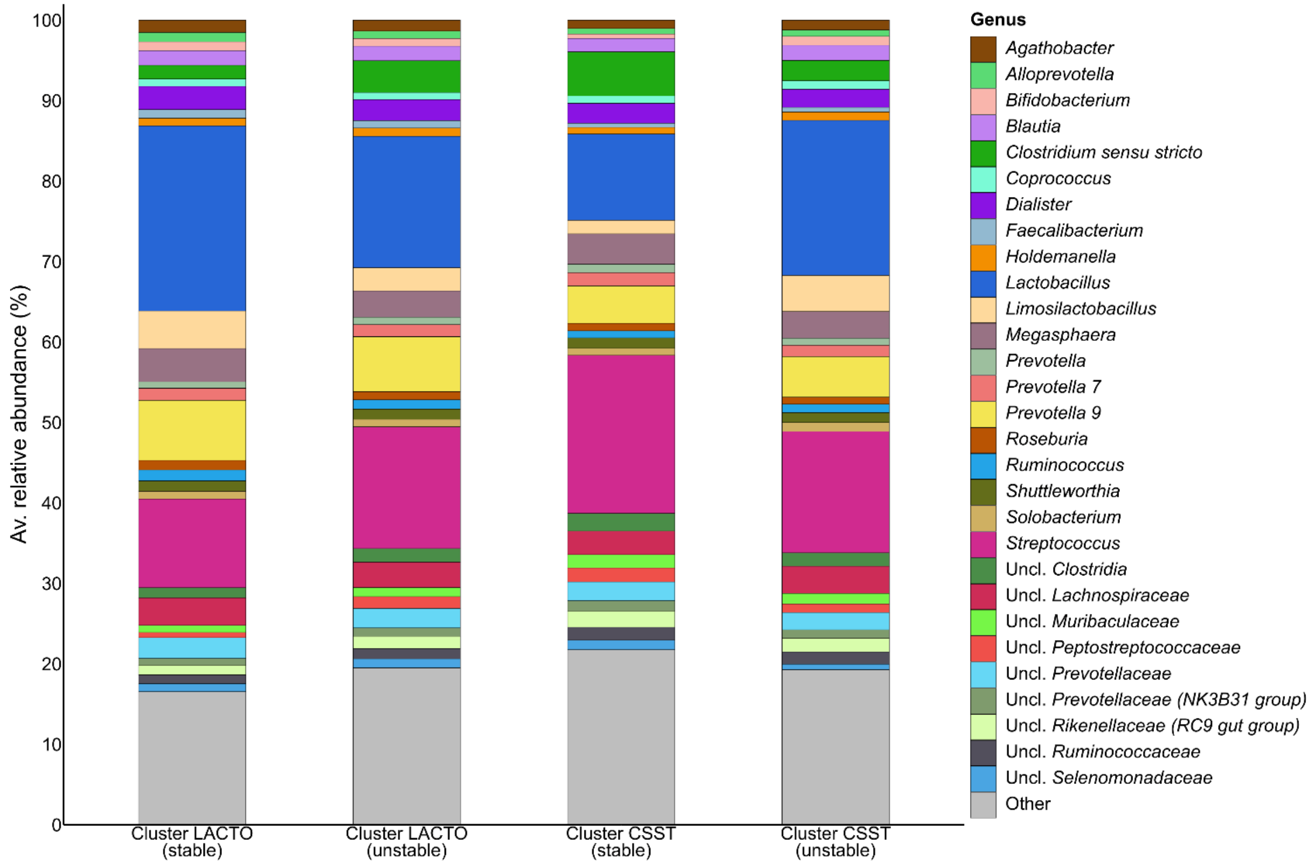

Figure S5. Microbial composition in the subgroups of the enterotype-like clusters. Comparison of the relative abundance at the genus level. Other contains genera with abundances < 1%.

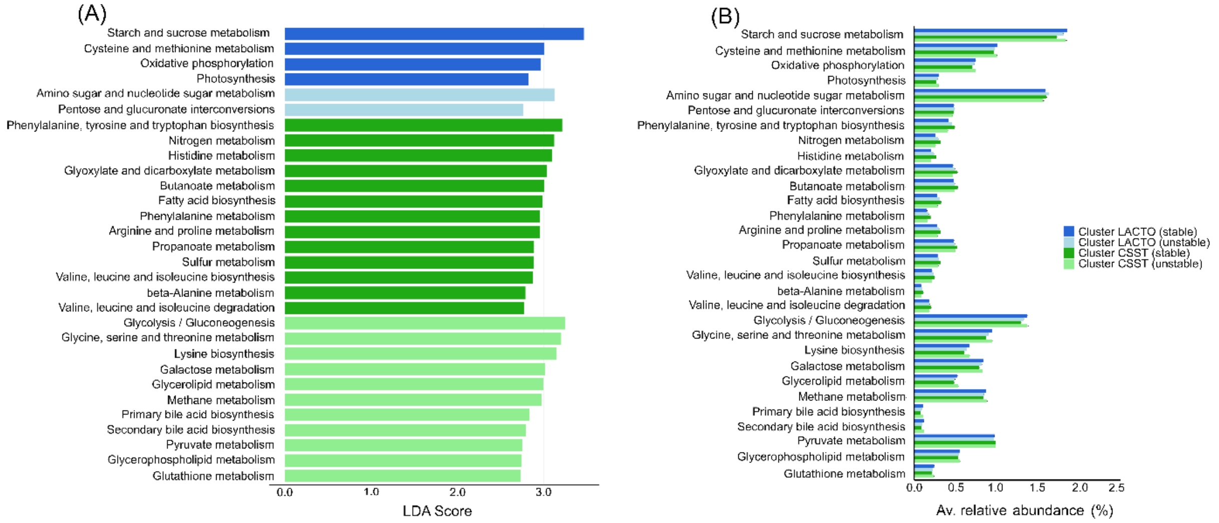

Figure S6. KEGG pathways belonging to “Amino acid metabolism”, “Carbohydrate metabolism”, “Lipid metabolism”, “Metabolism of other amino acids” and “Energy metabolism”. (A) Top 30 KEGG pathways identified by LefSe in each enterotype-like cluster and (B) respective averaged relative abundances both divided by subgroups.

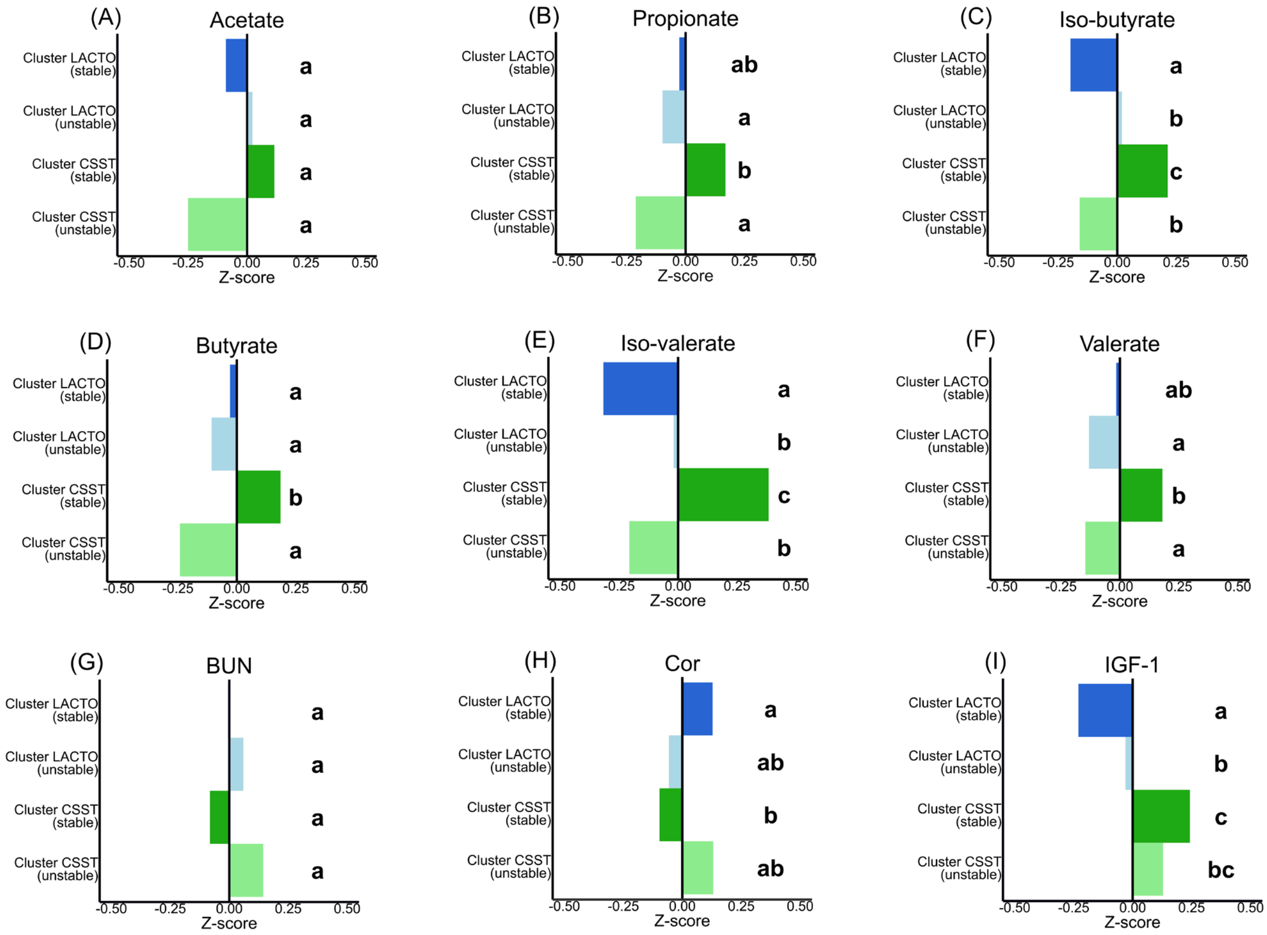

Figure S7. Comparison of the Z-scores of (A) acetate, (B) propionate, (C) iso-butyrate, (D) butyrate, (E) iso-valerate, (F) valerate, (G) BUN (blood urea nitrogen), (H) Cor (cortisol), (I) IGF-1 (insulin-like growth factor 1) between subgroups of the enterotype-like clusters.
Different letters indicate significant differences in the row data ($P < 0.05$).

1.2 Supplementary Tables

Supplementary tables are attached in a separated excel file.
